# The Endocannabinoid System and PPARs: Focus on Their Signalling Crosstalk, Action and Transcriptional Regulation

**DOI:** 10.3390/cells10030586

**Published:** 2021-03-07

**Authors:** Fabio Arturo Iannotti, Rosa Maria Vitale

**Affiliations:** Institute of Biomolecular Chemistry, National Research Council (ICB-CNR), Via Campi Flegrei 34, 80078 Pozzuoli (NA), Italy

**Keywords:** peroxisome proliferator-activated receptors (PPARs), endocannabinoid system (ECS), endocannabinoidome, plant cannabinoids, cannabinoid receptors, metabolism, neuroprotection

## Abstract

Peroxisome proliferator-activated receptors (PPARs) are a family of nuclear receptors including PPARα, PPARγ, and PPARβ/δ, acting as transcription factors to regulate the expression of a plethora of target genes involved in metabolism, immune reaction, cell differentiation, and a variety of other cellular changes and adaptive responses. PPARs are activated by a large number of both endogenous and exogenous lipid molecules, including phyto- and endo-cannabinoids, as well as endocannabinoid-like compounds. In this view, they can be considered an extension of the endocannabinoid system. Besides being directly activated by cannabinoids, PPARs are also indirectly modulated by receptors and enzymes regulating the activity and metabolism of endocannabinoids, and, vice versa, the expression of these receptors and enzymes may be regulated by PPARs. In this review, we provide an overview of the crosstalk between cannabinoids and PPARs, and the importance of their reciprocal regulation and modulation by common ligands, including those belonging to the extended endocannabinoid system (or “endocannabinoidome”) in the control of major physiological and pathophysiological functions.

## 1. PPAR Receptors Classification, Distribution, and Function

Peroxisome proliferator-activated receptors (PPARs) are a family of ligand-activated receptors/transcriptional factors composed by three distinct isoforms called PPARα (nuclear receptor subfamily 1 group C, NR1C1), PPARβ/δ (NR1C2), and PPARγ (NR1C3), each of which is encoded by independent genes in rodents and humans. Although co-expressed in several different types of organs and tissues, each isoform shows distinctive functional characteristics and ligand specificity [1,2]. Since their identification, PPARs have been recognized as sensing receptors for a variety of endogenous lipids (such as unsaturated, monounsaturated, and poly-unsaturated fatty acids) and natural exogenous compounds.

### 1.1. PPARα

PPARα is highly expressed in organs and tissues characterized by a high rate of fatty acid catabolism for energy production including liver, brown adipose tissue, endocrine tissues, gastrointestinal tract, cardiac and skeletal muscle. To a lesser extent, PPARα is present in the kidney, adrenal tissues, endothelial and immune (i.e. macrophages, monocytes, and lymphocytes) cells [3,4]. Additionally, PPARα has been found in specific brain areas where it exerts anti-inflammatory and neuroprotective actions (see next section) [5,6,7,8,9].

#### 1.1.1. Role of PPARα in Metabolism

PPARα plays a major role in metabolic homeostasis regulating lipid metabolism. Specifically, during the fed-to-fasted transition, PPARα drives the production of enzymes responsible for fatty acid oxidation (FAO) and the synthesis of ketone bodies from fatty acids in the liver. Thereby, PPARα works as a hub that integrates multiple metabolic signals to orchestrate the switch from glucose to fatty acid utilization for energy production [10,11]. Additionally, PPARα governs hepatic amino acid metabolism [12]. Studies on PPARα in other tissues including the heart, small intestine, skeletal muscle and brain have indicated that the role of PPARα in metabolic homeostasis is well conserved between different cell types [12,13,14,15]. Interestingly, *Ppar*α KO mice, under a normal dietary regimen, do not display pronounced anomalies in their phenotype. However, under fasting conditions or a high-fat diet, *Pparα* KO mice develop hypoglycemia and dyslipidemia characterized by excessive production of triacylglycerols [13,16,17]. Moreover, the lack of PPARα causes cardiac metabolic and contractile dysfunction, morphological and functional alterations in the brain in association with micro-/macro- vasculature dysfunctions [15,18,19]. In general, although the *Pparα* KO mouse model still shows unresolved aspects that are most likely attributable to the compensatory role of the other two PPAR isotypes, it allowed a deeper understanding of the role of PPARα in energy metabolism.

#### 1.1.2. Neuroprotective and Anti-Inflammatory Role of PPARα

Although all three PPAR isoforms are expressed in the nervous system during embryogenesis, only PPARβ/δ expression remains high in the brain, whereas PPARα and PPARγ expression decreases postnatally and remain restricted to the specific brain areas [20]. In particular, PPARα is expressed in basal ganglia, the reticular formation, some thalamic, mesencephalic and cranial motor nuclei and the large motoneurons of the spinal cord [5]. PPARα is expressed in dopamine neurons of the substantia nigra and spiny neurons of the dorsal striatum where it decreases dopaminergic transmission thus exerting an important control in behavioural responses [8,21,22]. The expression of PPARα is also reported in different subfields of the hippocampus of rodents, where it is also involved in the control of neuronal excitability and synaptic plasticity via cyclic AMP response element-binding protein (CREB) [23]. Moreover, PPARα is present in oligodendrocytes, microglia and astrocytes [24,25,26]. The studies reported above as well many others suggest that activation of PPARα in the brain initiates anti-oxidative and anti-inflammatory processes that confer neuroprotection. An effect also exerted by preserving the microvasculature activity [27]. The neuroprotective role of PPARα has been documented in numerous studies using several genetic and/or pharmacological models of neurodegenerative disorders including stroke, Alzheimer’s disease, Parkinson’s disease, traumatic brain injury, diabetic peripheral neuropathy, and retinopathy [7,9,22,28,29]. Besides its neuroprotective role, PPARα exerts important anti-inflammatory effects also in peripheral organs and tissues [3,30]. In this regard, Luisa et al., 2009, using an experimental model of inflammatory bowel disease, showed that *Pparα* KO mice compared to wild-type (WT) ones, have a deficient anti-inflammatory response [31]. Additionally, PPARα agonists have been reported to exert anti-inflammatory, antipyretic, anti-atherogenic and analgesic effects as demonstrated in experimental models of spinal cord trauma [28,32], neuropathic pain [33,34,35] and high-fat diet (HFD) induced atherosclerosis [36].

### 1.2. PPARγ

PPARγ is considered a master regulator of adipogenesis and is abundantly expressed in adipose tissue where it is primarily involved in fat and carbohydrate metabolism. Additionally, PPARγ exerts anti-inflammatory effects in several types of tissues and organs by repressing the expression and function of pro-inflammatory factors such as NF-κB and Nrf2/CREB and it is implicated in cancer and atherosclerosis [32]. The expression of PPARγ was also reported in other organs and tissues including the liver, skeletal muscle, spleen, heart, placenta, lung, ovary, and also brain (glial cells and neurons) [33,34].

#### 1.2.1. Role of PPARγ in Metabolism

In both rodents and humans, PPARγ is a master regulator of adipocytes differentiation as well as glucose and lipid metabolism [37]. Additional actions of PPARγ include the regulation of adipokines and inflammatory mediators expression, M1/M2 macrophage polarization, atherosclerosis and bone formation [38,39]. Genetic ablation of PPARγ in mice is lethal [40]. However, in an elegant study conducted by Gavrilova et al. [41], it was demonstrated that young mice with a PPARγ-deficient liver show a similar profile of wild-type (wt) mice. However, with ageing, LPPARγ Knock-Out (*Lpparγ*-KO) mice develop fat intolerance, increased adiposity, hyperlipidemia, and insulin resistance. Interestingly, when fed with a lipogenic diet even young LPPARγ-KO mice developed obesity. Other studies showed that mutant PPARγ mice show a complex metabolic phenotype including increased lean mass with organomegaly, hypermetabolism, hyperphagia, and lipoatrophy [42,43,44]. Moreover, Lüdtke et al. reported that PPARγ mutations lead to a familial form of lipodystrophy [45]. Therefore, these findings provide evidence that PPARγ is a key target to protect the liver as well as other organs and tissues from fat accumulation, insulin resistance, and uncontrolled inflammatory responses. Interestingly, the PPARγ gene is characterized by distinct mRNA isoforms due to alternative splicing of five exons at the 5′-terminal regions (A1, A2, B, C, and D). In particular, among the seven distinct isoforms identified so far, the most known are PPAR-γ1, -γ2, and –γ3. The PPARγ1 mRNA isoform is expressed in a wide range of organs and tissues including those aforementioned and also in the immune cells (e.g., monocytes/macrophages). On the contrary, the expression of PPARγ2 mRNA seems restricted to adipose tissue, and PPARγ3 is localized in macrophages, colon, and adipose tissue [37,46,47,48,49]. Of note, in 2004 Zhang et al. showed that PPARγ2 deficient mice (*PPARγ* ^-/-^) have an impaired fat metabolism and insulin resistance, thus demonstrating the central role of PPARγ2 in adipogenesis [14]. It is important to recall that different PPARγ isoforms may be responsible for unique tissue-specific biological effects in response or not to endogenous and/or exogenous ligands. 

#### 1.2.2. Neuroprotective and Anti-Inflammatory Role of PPARγ

PPARγ, similarly to PPARα, in the brain exerts anti-inflammatory and neuroprotective effects through the inhibition of NFkB, AP-1, STATs, and iNOS [50]. This finding has been confirmed in several animal models of Alzheimer’s and Parkinson’s disease. Moreover, the synthetic PPARγ agonist pioglitazone was shown to extend the survival of SOD1-G93A, a mouse model of Amyotrophic lateral sclerosis (ALS), by delaying the onset of the disease and preventing the death of motor neuron cells [51]. Activation of PPARγ has been shown to exert neuroprotective effects also by preventing astro- and microglial activation [52]. Additionally, PPARγ has been shown to downregulate the expression of pro-inflammatory factors and free radical production in various intestinal disorders including colon cancer, gastritis and irritable bowel syndrome, and to also exert anti-nociceptive effects against somatic pain, allergic and skin diseases [53,54,55].

### 1.3. PPARβ/δ

PPARβ/δ is ubiquitously expressed and its biological functions mainly overlap with those of the other two PPAR isoforms. Recently, the expression of PPARβ/δ was demonstrated in neurons, astrocytes, oligodendrocytes, and also, in microglial cells [56,57]. Several studies demonstrate that PPARβ/δ plays an important role in inflammatory processes and, due to its proangiogenic and anti-/pro-carcinogenic properties, it is considered a therapeutic target for treating metabolic syndrome, dyslipidemia, diabetes while the role of PPARβ/δ on cancerogenesis is still debated [58,59]. In the brain, PPARβ/δ could act as a protective factor for counteracting inflammation and promoting antioxidant mechanisms [60]. Additionally, PPARβ/δ is known to control cell cycle, proliferation, differentiation and also inhibit cell death by promoting the expression of ILK and PDK1 in many types of cells [61,62]. However, compared to the other two isoforms, the role of PPARβ/δ in inflammation still needs to be fully elucidated. For example, at peripheral level, stimulation of PPARβ/δ was shown to promote the inhibition of inflammatory response in streptozotocin-induced diabetic nephropathy and vasculopathy [63,64]. In contrast, other studies report that PPARβ/δ appears to promote inflammation in other contexts. This latter evidence was observed in murine models of skin disorders (i.e. psoriasis) and arthritis [65].

## 2. Structure of PPARs

**Figure 1:**
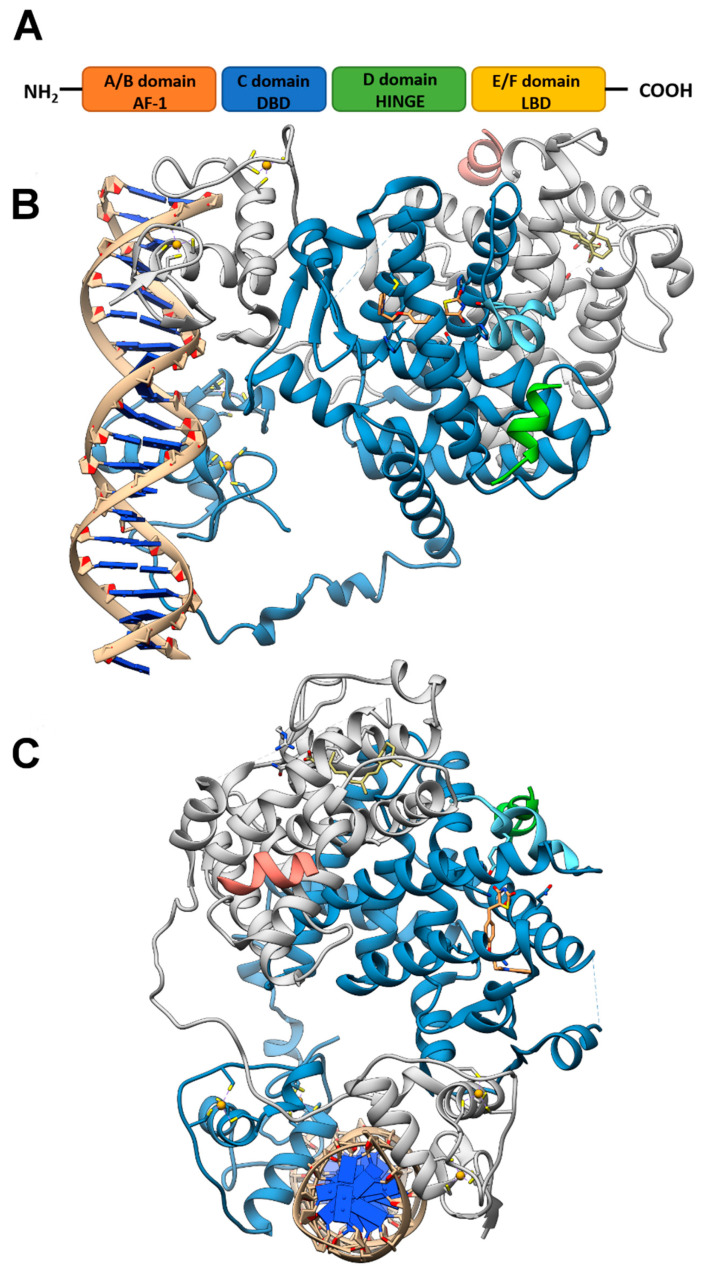
PPAR domains and 3D model of their interaction with RXRα and target DNA (**A**) Schematic representation of the PPAR domain organization and (**B**,**C**) orthogonal views of the x-ray structure of PPARγ/RXRα heterodimer in complex with DNA and coactivator peptides (PDB id: 3DZY). PPARγ is colored in slate blue with helix H12 in sky blue, RXRα in gray, the coactivator peptides in green and salmon and DNA in tan. The ligands rosiglitazone and 9-cis-retinoic acid are shown in rose brown and khaki, respectively, while the Zn(II) ions are in gold. Nitrogen, oxygen and sulfur atoms are colored in blue, red and yellow, respectively. This figure has been realized with the Chimera v.1.14 program.

**Figure 2:**
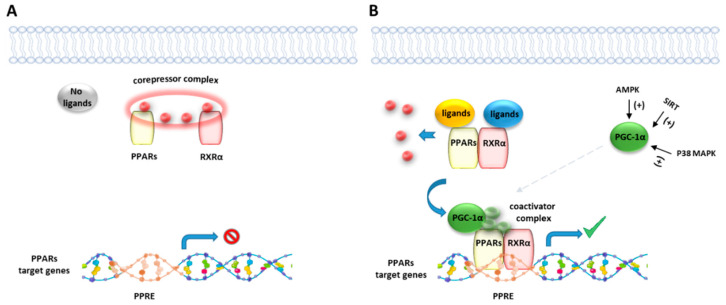
Representative illustration of PPARs activating pathways. (**A**) In absence of ligand binding, the nuclear corepressor complex prevents PPAR/RXR binding to their DNA binding element, called PPAR response element (PPRE). (**B**) In presence of a ligand, PPAR/RXR dimer binds to the PPRE of target genes and regulates the transcription of target genes. Peroxisome proliferator-activated receptor gamma coactivator 1-alpha (PGC-1α) is a transcriptional coactivator of the PPAR/RXR complex, known to be activated by intracellular factors such as AMPK, SIRT and MAP kinases.

Like other nuclear receptor superfamily members, PPAR structure is organized in four domains, named A/B, C, D, and E/F: (i) the N-terminal A/B domain contains the ligand-independent activation function 1 (AF1) responsible for the transcriptional activation; (ii) the C domain consists in the DNA-binding domain (DBD), formed by two zinc-finger motifs responsible for the binding to peroxisome proliferator response elements (PPREs) within the promoter regions of target genes; (iii) the D domain (hinge domain) modulates the ability of the receptor to bind DNA and it is involved in corepressors binding; (iv) C-terminal E/F domains correspond to the ligand-binding domain (LBD) which contains the ligand-binding pocket (LBP), the ligand-dependent activation function (AF-2) and regions important for the heterodimerization with the retinoid X receptor (RXR) [66,67] (Figure 1A). The PPAR-RXR heterodimer is a prerequisite for PPARs to bind the PPREs in the promoter region of target genes. They act as permissive heterodimers, being activated either by PPAR or RXR ligands. However, the simultaneous presence of both ligands gives rise to a synergistic response [68]. When PPAR-RXR heterodimers are not bound to a ligand, they act as repressors through association with corepressor complexes (Figure 1B,C; see also Figure 2). The ligand binding to LBP induces conformational changes in the AF2 region, facilitating the recruitment of coactivators and the release of corepressors [69]. In detail, canonical agonists within the LBP form a network of hydrogen bonds stabilizing the conformation of a short helix, the helix12 (H12), which in turn allows the interaction of coactivators with the AF-2 surface. However, PPAR ligands can also bind to distinct sub-regions or allosteric sites and activate these receptors through H12-independent mechanisms to elicit a partial agonist activity [70]. Besides the ligand-dependent mechanism, other mechanisms are known to regulate the transactivation and trans-repression activity of PPARs. In particular, the PPAR activity is governed by various post-translational modifications including phosphorylation, SUMOylation, ubiquitination, acetylation, and O-GlcNAcylation [71].

## 3. The Endocannabinoidome, Including and beyond the Endocannabinoid System

**Figure 3:**
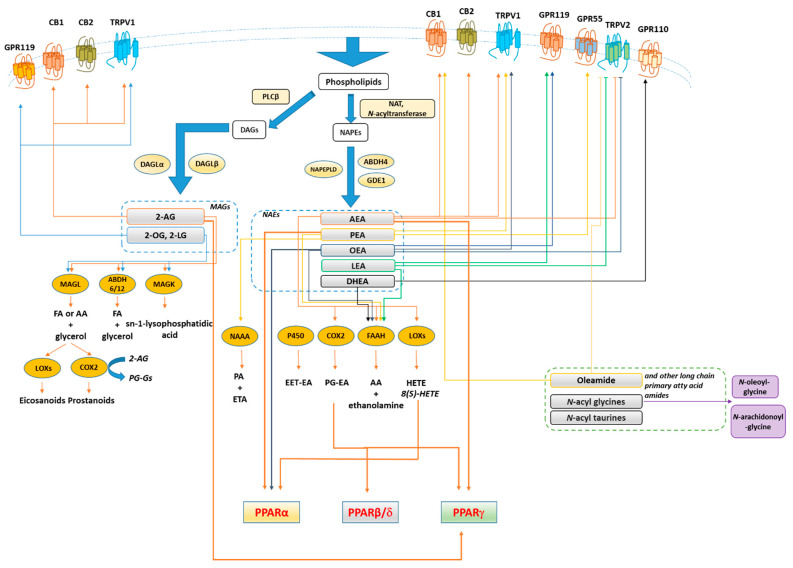
Synthesis, inactivation, receptors and main metabolic functions of endocannabinoidome mediators. Thick arrows denote the biochemical reactions and functional connections underlying endocannabinoidome mediator action. Not shown, for the sake of clarity, are the negative effects exerted by almost all unsaturated *N*-acyl-amides tested so far on T-type (Ca_v_3) Ca^2+^ channels. Blunted arrows denote inhibition. Abbreviations: 2-AG, 2-arachidonoylglycerol; 2-LG, 2-linoleoyl glycerol; 2-OG, 2-oleoyl glycerol; 5/12/15-LOX, 5/12/15-lipoxygenase; 15 HAEA, 15(S)-HETE Ethanolamide; AA, arachidonic acid; ABH4/6/12, αβ-hydrolase 4/6/12; AEA, Anandamide; CB1/2, cannabinoid receptor 1/2; COX2, cyclooxygenase 2; DAG, diacylglycerols; DHEA, *N*-docosahexaenoyl-ethanolamine; EET-EA, epoxyeicosatrienoic acid ethanolamide; FA, fatty acid; FAAH, fatty acid amide hydrolase; FA, free fatty acids; GDE1, glycerophosphodiester phosphodiesterase 1; GPR, G-protein-coupled receptor; LEA, *N*-linoleoyl-ethanolamine; MAGL, monoacylglycerol lipase; MAG, monoacylglycerols NAAA, *N*-acylethanolamine-hydrolysing acid amidase, NAPE, *N*-acyl-phosphatidylethanolamine; NAPEPLD, *N*-acyl-phosphatidylethanolamine-specific phospholipase D; NAT, *N*-acyltransferase; OEA, *N*-oleoyl-ethanolamine; P450, cytochrome p450 oxygenases; PEA, *N*-palmitoylethanolamine; PGE2, Prostaglandin E2; PLCβ, phospholipase Cβ; PPARγ, peroxisome proliferator-activated receptor γ; PPARα, peroxisome proliferator-activated receptor α; SEA, *N*-stearoyl-ethanolamine; TRPV1, transient receptor potential vanilloid type-1 channel; TRPV2, transient receptor potential vanilloid type-2 channel.

The endocannabinoid system (ECS) refers to a large group of lipid mediators, enzymes, and receptors largely distributed throughout the mammalian body. The major component of ECS are 1) two main lipid signaling molecules, the eCBs anandamide (*N*-arachidonoyl-ethanolamine, AEA) and 2-arachidonoyl-glycerol (2-AG); 2) a large number of biosynthesizing and inactivating enzymes of which the most studied ones are ABDH4, GDE-1, NAPE-PLD and PTPN22 for the biosynthesis of AEA from *N*-arachidonoyl-phosphatidylethanolamine; FAAH for its degradation to arachidonic acid (AA) and ethanolamine; DAGLα and DAGLβ for the biosynthesis of 2-AG from sn-2-AA-containing diacylglycerols, and ABDH6, ABDH12 and MAGL for its degradation to AA and glycerol; 3) two main metabotropic receptors responsive to AEA and 2-AG, named cannabinoid receptor of type-1 (CB1) and type 2 (CB2) [72,73,74] (Figure 3). Beyond its classical definition, numerous studies revealed that many other *N*-acyl-ethanolamines (NAEs) including (i) *N*-palmitoylethanolamine (PEA), (ii) *N*-oleylethanolamine (OEA), (iii) *N*-linoleylethanolamine (LEA), (iv) *N*-stearoylethanolamine (SEA), DHEA and v) *N*-docosahexaenoyl-ethanolamine; monoacylglycerols (2-LG, 2-linoleoyl glycerol; 2-OG, 2-oleoyl glycerol); *N*-acyldopamines/taurines/serotonins together with G-protein coupled receptors (GPR55, GPR119, GPR18), ion channels (TRPV1 and TRPV2), metabolic enzymes (i.e. lipoxygenase LOX, cyclooxygenase COX, and cytochrome P450) as well as their related metabolites (i.e eicosanoids) are structurally and functionally related to the ECS activity. Moreover, 2-AG was found to act as a direct modulator of the GABAA receptor [75]. Therefore, these discoveries led to expanding our view of the ECS and to look at it as the ‘endocannabinoidome’ (Figure 2).

## 4. PPARs Ligands

After the discovery of all three PPARs isoforms in the early 90s, a large number of natural and synthetic ligands have been identified. The best known synthetic ligands of PPARα are fibrates (e.g., clofibrate, fenofibrate, and bezafibrate), a class of drugs used to reduce the risk of cardiovascular disease in patients with dyslipidemia due to their ability to lower plasma triglyceride levels and elevate HDL cholesterol. PPARγ is the molecular target of glitazones such as pioglitazone and rosiglitazone, which are approved drugs for diabetes. Moreover, many other synthetic dual and/or pan- PPARs agonists (e.g., like muraglitazar and tesaglitazar) have been tested in the treatment of obesity, dyslipidemias and type 2 diabetes or for the treatment of hypertension [76,77,78,79,80]. To date, several endogenous PPAR ligands have been reported so far, including fatty acids (e.g., docosahexaenoic and eicosapentaenoic acid), eicosanoids (e.g., leukotriene B_4_ stimulates PPARα, and prostaglandin PGJ_2_ activates PPARγ). In this context, we focus on the role of (endo)cannabinoid molecules as PPAR ligands and the functional interaction between PPARs and enzymes and receptors related to the endocannabinoid system.

### Cannabinoids and Cannabinoid-Like Molecules as PPARs Modulators

**Figure 4:**
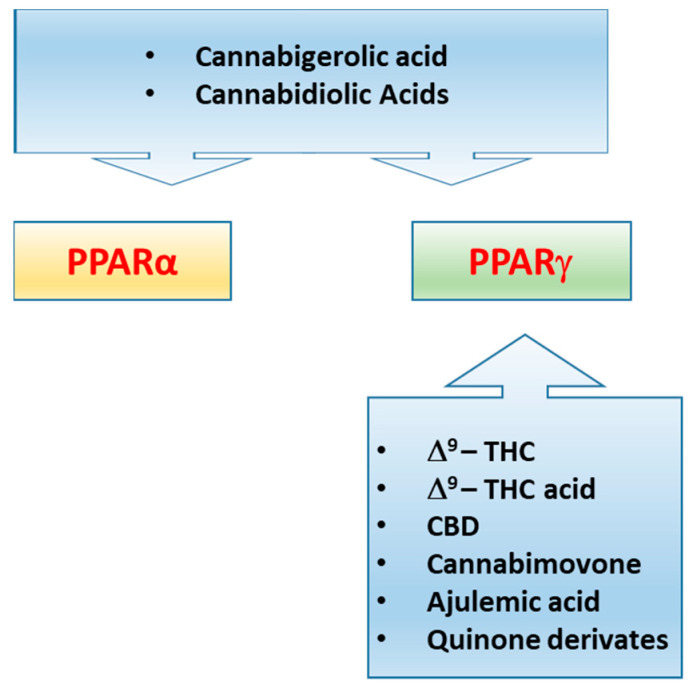
Representative illustration showing the different ability of some plant cannabinoids, ajulemic acid and quinone derivatives to activate PPARα and/or PPARγ.

It has been demonstrated that PPARs, mainly the isoforms α and γ, concur in mediating the metabolic and anti-inflammatory effects of (endo)cannabinoid molecules, towards which they exhibit a different selectivity profile. In this regard, it has been demonstrated that synthetic and plant-derived cannabinoids attenuate neuroinflammation and neurodegeneration in animal models of acute or chronic neurodegenerative disorders through activation of cannabinoid receptors and PPARγ pathway [81,82,83,84]. While natural and synthetic phytocannabinoids including Δ^9^-tetrahydrocannabinol (Δ^9^-THC), Cannabidiol (CBD), Δ9-THC acid, ajulemic acid, quinone derivatives [85,86,87,88,89], and the recently identified cannabimovone [90] are PPARγ agonists, the acidic derivatives cannabigerolic and cannabidiolic acids exhibit a dual agonist profile [91] (Figure 4). The endocannabinoid anandamide (AEA) activates both PPARα [92] and PPARγ [93], albeit its efficacy and potency toward PPARα are higher in comparison to PPARγ. The endocannabinoid-like-compounds OEA and PEA, structurally related to AEA, do not bind endocannabinoid receptors with high affinity but exert their metabolic and anti-inflammatory effects mainly through PPARα activation [94,95,96]. A comparative study carried out on a series of ole-acylethanolamines, such as PEA, SEA, OEA, AEA, LEA, DHEA and eicosapentaenoyl-ethanolamide (EPEA) showed that all the NAEs activate PPARα, albeit with different potency, and OEA and LEA were the most potent [97]. The endocannabinoid 2-arachidonyl glyceryl ether (2-AGE, noladin ether) and virodhamine (O-arachidonoyl ethanolamine; O-AEA) bind to PPARα with a rank order noladin ether>anandamide>virodhamine (EC50 values 10–30 µM), although they show less efficacy than anandamide at 10 µM [98]. 2-AG and its non-hydrolyzable analogue, 2-AGE, were shown to activate PPARγ, through which 2-AG promotes the suppression of interleukin (IL-2) [99]. However, the dependence on COX-2 metabolism of 2-AG for the suppression of IL-2 suggested the active role of a 2-AG metabolite in PPARγ activation, identified by Raman et al. [100] as the 15d-PGJ2-glycerol ester, while Kozak et al. [101] showed that lipoxygenase metabolism of 2-AG resulted in the release of the PPARα agonist 15-hydroxyeicosatetraenoic acid glyceryl ester (15-HETE-G). The 12/15-LOX arachidonic acid metabolites 12-HETE and 15-HETE increase the transcriptional activity of PPARγ in primary cortical neurons, eliciting neuroprotection through the inhibition of the inducible NF-kB, NO synthase, and COX-2, a suppressive effect reversed by the PPARγ antagonist GW9662 [102]. Yu et. al showed that arachidonic acid (AA), but not its precursor AEA, activates PPARβ/δ, unveiling a dual role for the fatty acid-binding protein FABP5 in AA-mediated activation of PPARβ/δ, since it promotes both the hydrolysis of AEA by FAAH and the translocation of the enzymatic product arachidonic acid to the nucleus, thus enabling PPARβ/δ activation by AA [103]. The cannabinoid-like oleamide also was shown to activate PPARβ/δ, along with PPARα and PPARγ [104]. Further details on cannabinoid molecules as PPAR ligands is beyond the scope of the present review since this topic has been extensively reviewed by Pistis & O’Sullivan [105].

## 5. Cross-Talk between PPARs and Metabolizing Enzymes Related to Endocannabinoids

A pharmacological strategy to promote the activity of PPARs is to elevate the level of endogenous modulators. In this view, Mazzola et al. [106], using a passive-avoidance task in rats, demonstrated that the pharmacological inhibition of FAAH enzyme by URB597 recapitulates the effects of selective PPARα agonist administration on memory enhancement, by increasing the level of the endogenous PPARα agonists *N*-acylethanolamines (NAEs) such as AEA and OEA. A similar strategy has been pursued with NAAA inhibitors to increase the endogenous levels of PEA [107], which, besides directly activating PPARα, enhances the endogenous tone of *N*-acylethanolamines (NAEs) through the down-regulation of FAAH expression and activity [108], thus explaining, at least in part, its entourage effect. Besides the indirect effects of FAA and NAAA inhibitors, PPARs have been shown to directly regulate the expression level of some endocannabinoid metabolizing enzymes such as cyclooxygenases and lipoxygenases. Cyclooxygenase-2 (COX-2) catalyzes the biosynthesis of prostaglandins (PGs) from arachidonic acid. Among PGs, the PGD2 metabolite 15-deoxy-D^12,14^ PGJ2 (15d-PGJ2) has been identified as a potent agonist PPARγ. Several reports have shown that COX-2 is down-regulated upon PPARγ [109] stimulation, while the epidermal growth factor (EGF), a potent activator of COX-2, induces an enhanced COX-2 expression and a decrease of PPARγ mRNA levels. This down-regulation is linked to COX-2 signalling since it is reversed by NS-398, a selective COX-2 inhibitor [109]. The ability of PPARγ agonists to down-regulate COX-2 expression has been observed in breast cancer cells, macrophages, and cervical cancer cell lines. However, the findings that PPARγ ligands can also up-regulate COX-2 expression in several cell lines [110] is suggestive of a tissue-specific cross-regulation. Moreover, the positive or negative modulation of COX-2 expression by PPARγ ligands could be either PPARγ- dependent or independent [109]. Lipoxygenases (LOXs) are enzymes that catalyze the conversion of polyunsaturated fatty acids, mainly arachidonic and linoleic acids, to hydroxy fatty acid and are classified according to the carbon atom they peroxygenase on a particular substrate. In humans, two forms of 15-LOXs, namely 15-LOX-1 and 15-LOX-2, occur, leading to 13-S-hydroxyoctadecadienoic acid (13-S-HODE), respectively, which activate PPARγ. It was found [111] that an inverse relationship exists between 15-LOX-2 and PPARγ expression levels in normal vs tumour cells, suggestive of the occurrence of a feedback mechanism regulating the expression levels of these proteins. Indeed, the overexpression of PPARγ1 and PPARγ2 was shown to dose-dependently downregulate 15-LOX-2 expression in prostate epithelial cells, effect increased by treatment with the 15-S-HETE [48]. Similarly, the overexpression of PPARγ1 downregulates 15-LOX-2 in normal epithelial cells from breast or lung tissues, effect enhanced by 15-S-HETE. The feedback regulation of PPARγ by 15-LOX-2 was determined [111] by overexpressing 15-LOX-2 in tumor cells with high levels of PPARγ and observing a concentration-dependent downregulation of PPARγ with increasing amounts of 15-LOX-2, an effect enhanced by coincubation with 15-S-HETE.

## 6. Cross-Talk between PPARs and GPCRs

PPARs have been shown to modulate the expression level of G-protein coupled receptors (GPCRs) linked to cell metabolism, inflammation and cancer. In turn, the activation of certain GPCRs was shown to modulate the expression levels of PPARs. GPR120 is the cognate receptor for omega 3 long-chain fatty acids and it is involved in insulin sensitization and inflammation. It is highly expressed in mature adipocytes, where it stimulates adipocyte differentiation [112,113,114,115]. Omega 3 fatty acids activate PPARγ through both direct binding [116,117] and the GPR120-mediated PI3K pathway [118]. Silencing or overexpression of GPR120 in adipocytes was shown to inhibit or enhance the expression of PPARγ, respectively [119]. Silencing of PPARγ inhibits the induction of microRNA-143 by GPR-120 in adipocytes, suggesting the involvement of PPARγ in its GPR120-mediated transcriptional activation [119]. The transcriptome analysis in human adipocytes treated with the PPARγ agonist rosiglitazone showed that it induces the expression of the anti-lypolitic GPR81 and GPR109A in human and murine adipocytes as well as the human-specific GPR109B in differentiated adipocytes. The reduced expression of PPARγ by siRNA-mediated knockdown or the use of the PPARγ-specific antagonist GW9662 was shown to reduce the levels of both GPR81 and GPR109A, indicating that PPARγ directly regulate the expression of these receptors, although responsive PPRE elements were found only within the promoter region of Gpr81 gene. Thus, it remains unclear whether PPARγ controls the transcriptional activity of the GPR109A gene by direct mechanisms [120]. Evidence of cross-talk between GPR109A and PPARγ was also reported by, Knowles et al. [121], who showed that Niacin, a GPR109A agonist, induces transcriptional activity and activation of PPARγ in macrophages through the stimulation GPR109A-mediated of the prostaglandin synthesis pathway. Additionally, PPARγ upregulates the expression of both the short-chain free fatty acid receptor GPR43, another anti-lipolytic G-protein coupled receptor expressed in adipocytes, and the long-chain fatty acid receptor GPR40 in pancreatic β-cells [122]. Besides the transcriptional effects on GPRs involved in cell metabolism, PPARγ was shown to downregulate the chemokine receptor CXCR4, which is highly expressed in cancer cell metastasis [123].

## 7. Crosstalk between PPARs and Cannabinoids Receptors

### 7.1. CB1R and PPARα

**Figure 5:**
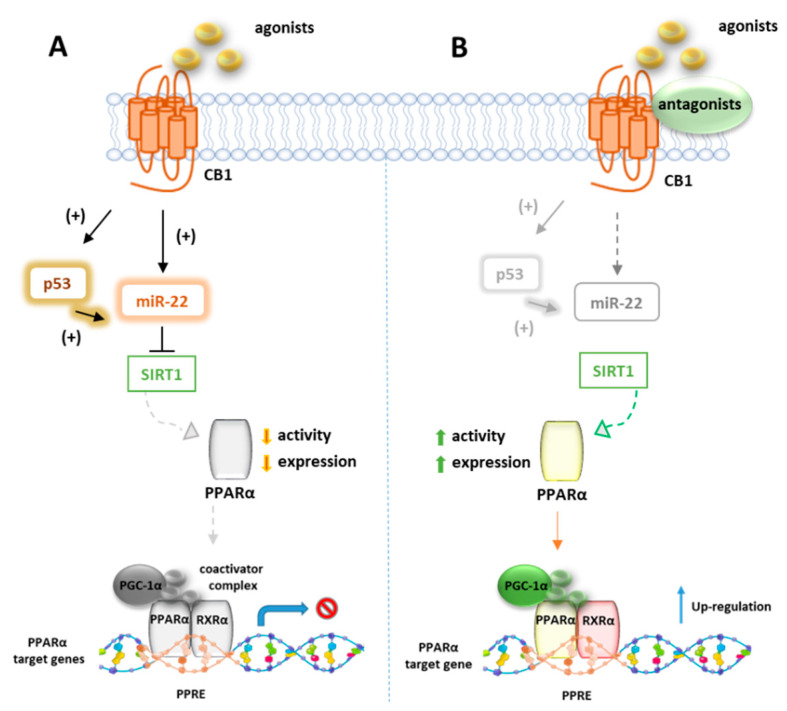
Representative mechanism of PPARα gene regulation by CB1 receptors. (**A**) In absence of CB1 ligands, p53 and miRNA22 suppress the activity of SIRT1 which positively regulates PPARα. (**B**) In presence of CB1 agonists, p53 and miRNA22 are inhibited and SIRT1 activates PPARα which forms a heterodimer with RXR, to bind PPRE elements of target genes.

Azar et al. in a recent paper disclosed the existence of a molecular link between CB1R and p53/miR-22/SIRT1/PPARα signaling in hepatocytes [124]. Using unbiased bioinformatics techniques and combined in vitro and in vivo experiments, they found that PPARα is evolutionarily coevolved with CB1R and is negatively modulated by CB1R in hepatocytes since ACEA can downregulate both mRNA and protein levels of PPARα as well as its targeted genes. These effects were reversed by the CB1R antagonist AM6545, which conversely induces the hepatic expression of PPARα and upregulates its target genes in WT diet-induced obese mice (DIO) after 7-day treatment. The authors also provided evidence that AM6545 is unable to directly interact with PPARα, further corroborating the finding that hepatic PPARα is controlled by the CB1R. In DIO mice treated with AM6545, a robust upregulation of the eCBs PPARα ligands AEA, AA, OEA, and PEA was observed at hepatic level, probably due to an increased synthesis rather than the degradation rate since AM6545 is ineffective toward FAAH or MAGL. The increased level of these endogenous ligands is supportive of the correlation between the CB1R blockage and the increased hepatic activation of PPARα. Next, the authors found that the expression and function of SIRT1, a key modulator of hepatic PPARα activity, is regulated by CB1R since its activation reduces both the expression and the deacetylase activity of SIRT1 in hepatocytes, effect reversed by AM6545 (Figure 5). Since the abnormal expression of microRNA (miR-22) is reportedly involved in the regulation of both SIRT1 and PPARs expression or activity in various tissues, a miR sequence profiling of liver tissues from the DIO mice treated with AM6545 or vehicle was performed. The authors found that the expression level of miR-22, which downregulates SIRT1 and PPARα in hepatocytes, is significantly reduced by AM6545 or CB1R ablation (Figure 5). Moreover, the effect of ACEA in reducing the expression of SIRT1 and PPARα is impaired by a miR-22 inhibitor, while AM6545 failed to reverse their CB1R-induced downregulation in presence of a mimic-miR-22. Finally, the transcription factor p53, known to induce a change in miR expression and to be regulated by CB1R, increases its activity in hepatocytes after ACEA treatment, an effect abolished by AM6545 or genetic ablation of the CB1R. The link between p53 and miR-22 was demonstrated by using DOX, a known activator of p53, which was found to increase the expression of miR-22 while the involvement of p53 in CB1R-induced miR-22 activation was confirmed by using a reversible transcriptional inhibitor.

### 7.2. CB1R and PPARγ

The endocannabinoid system has a well-established role in the regulation of energy homeostasis through the activation of CB1R [72,73,125]. Numerous studies show that the ECS is overactive in human obesity as well as animal models of genetic and diet-induced obesity [74,126] and thus the pharmacological blockade or genetic ablation of CB1 receptor prevent the risk of obesity and diabetes [127,128]. CB1R is not expressed in undifferentiated preadipocytes but became up-regulated in the early stage of differentiation, while CB2R is expressed in preadipocytes but is down-regulated at an undetectable level upon differentiation [129]. When the cells are differentiated in presence of the PPARγ agonist rosiglitazone, a down-regulation in CB1R and up-regulation in FAAH expression is observed in differentiated adipocytes, suggestive of a reduction in endocannabinoid tone, in agreement with the ciglitazone-induced decrease of 2-AG levels in partially differentiated adipocytes, observed by Matias et al [130]. On the contrary, the stimulation of preadipocytes in the early phase of adipocyte differentiation with the non-selective cannabinoid agonist WIN55,212 induces a significant expression of PPARγ, an effect no longer observed in fully differentiated cells [129]. The same authors also showed an insulin-mimetic action CB1R-mediated of endocannabinoids on glucose uptake, involving PI3-kinase and the intracellular calcium influx. The increasing of PPARγ expression using WIN55,212 was also observed by Fakhfouri et al. [82], investigating the protective effect of this compound in the beta-amyloid-induced neurodegeneration in rat hippocampus. They observed that the effect on the transcriptional activity of WIN55,212 was partially inhibited with the CB1R antagonist AM251 or the PPARγ antagonist GW9662 but not with the CB2R antagonist SR144528, suggestive of both direct and CB1R-mediated effect on PPARγ signaling [82]. The lipoaminoacid *N*-Oleoylglycine (Olgly) was found to stimulate 3T3-L1 adipogenesis and to increase the expression level of PPARγ through the activation of the CB1 receptor and enhancement of insulin-mediated Akt signaling. Interestingly, Olgly also increases the expression level of CB1R mRNA, whereas the inhibition of CB1R by the antagonist SR141716 abolishes the effects of OLGly Olgly on lipid accumulation and PPARγ expression as well as the increment Olgly-dependent of p-Akt/Akt and p-FoxO1/FoxO1 ratio [131], confirming the link between CB1R activation and Akt signaling. Du et al. [132] showed that the 2-AG induced resolution of neuroinflammation in response to pro-inflammatory insults is mediated by the CB1R-dependent expression of PPARγ. In particular, the suppression of NF-kB-p65 phosphorylation, COX-2 expression, and excitatory synaptic transmission in response to proinflammatory IL-1β and LPS were inhibited by the selective PPARγ antagonist GW9662, in hippocampal neurons cultures and the effects of 2-AG were mimicked by the PPARγ agonists 15d-PGJ2 and rosiglitazone. Conversely, Lin et al. [133] demonstrated that in streptozotocin-induced diabetic rats, hyperglycemia causes glomerular hypertrophy and fibrosis with the concomitant increased expression level of inflammatory cytokines and CB1R and reduced PPARγ2 signaling. Knockdown of PPARγ2 mimicked the promotional effects of high glucose on CB1R signaling, suggesting that CB1R negatively regulates PPARγ2 signaling in mesangial cell cultures. Indeed, PPARγ2 signaling was restored by using CB1R antisense oligonucleotides or inverse agonist AM251. The same effect is obtained with the PPARγ agonist rosiglitazone, which increased PPARγ2 expression level and counteracted the hyperglycemia-induced enhancement of CB1 expression, inflammation, and glomerular fibrosis in diabetic animals.

### 7.3. CB2R and PPARγ Coactivator 1α (PGC-1α)/PPARγ

The transcriptional coactivator PGC-1α enhances the ability of PPARs and other nuclear receptors to promote gene transcription [134] in particular genes linked to fatty acid oxidation, metabolism, and inflammation, and it is upregulated by NAD+-dependent histone deacetylase sirtuin 1 (SIRT1). SIRT1 activity is increased through different pathways, such as glucose restriction, AMP-activated protein kinase (AMPK) activation, and cAMP response element-binding protein (CREB) activation [135,136]. Zheng et al. [137] showed that the selective CB2R agonist trans-caryophyllene, a bicyclic sesquiterpene isolated from cannabis also known as beta-caryophyllene (BCP), stimulates the activity of SIRT1 in skeletal muscle cells by increasing the phosphorylation of CREB, which leads to an enhanced level of PGC-1a deacetylation and in turn to a higher expression of the genes linked to fatty acid oxidation. The effect of BCP is mediated by CB2R since it is lost when the expression of CB2R in C2C12 myotubes is inhibited with CB2R siRNA. Thus, CB2R contributes to lipid homeostasis by increasing the rate of fatty acid oxidation through the activation of the SIRT1/PGC-1α pathway. It has been also reported that BCP improves anxiety, memory, and depression by modulating PGC-1α/BDNF pathway in a CB2R-dependent manner [138]. BCP has been shown to ameliorate arthritis by increasing PGC-1α and PPARγ expression in human articular chondrocytes and this effect is reverted by the CB2R antagonist AM630 [139]. The modulation of PGC-1α CB2R-mediated it has been also reported for the CB2R agonist AM1241, which was shown to reduce microglial inflammation by promoting PGC-1α-induced mitochondrial biogenesis in N9 microglial cells [140].

### 7.4. CB2R and PPARα

**Figure 6:**
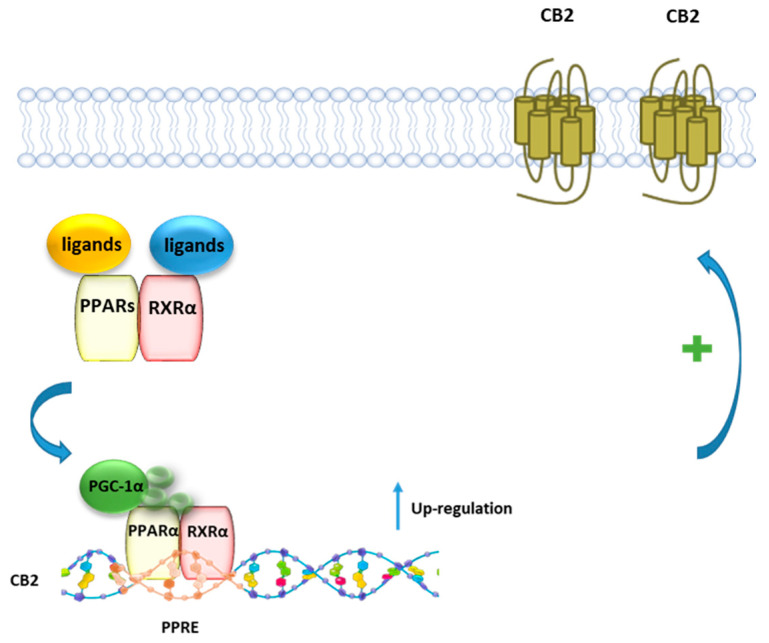
Representative mechanism of PEA and canonical PPARα agonists in regulating the transcriptional activity of CB2 gene. In presence of a ligand, PPARα forms a heterodimer with RXR, which binds to the PPRE of the CB2 gene, leading to an increased expression of CB2 protein.

Using a combined pharmacological, biochemical, and bioinformatics approach, it was shown that PEA, as well as the PPARα selective agonist GW7647, enhances CB2R expression via PPARα activation in both rat microglia and human macrophage cells [26] (Figure 6). The involvement of PPARα was demonstrated using either the PPARα antagonist GW6471, or PPARα silenced cells, where the incubation with PEA or GW7647 failed to increase CB2R expression. Moreover, it was shown that after stimulation with PEA, PPARα regulates the transcription of the gene encoding for CB2R *Cnr2* through the high-affinity binding to a specific region in the *Cnr2* gene, identified by a bioinformatic approach.

## 8. Crosstalk between PPARs and TRP Channels

### 8.1. PPARα and TRPV1

**Figure 7:**
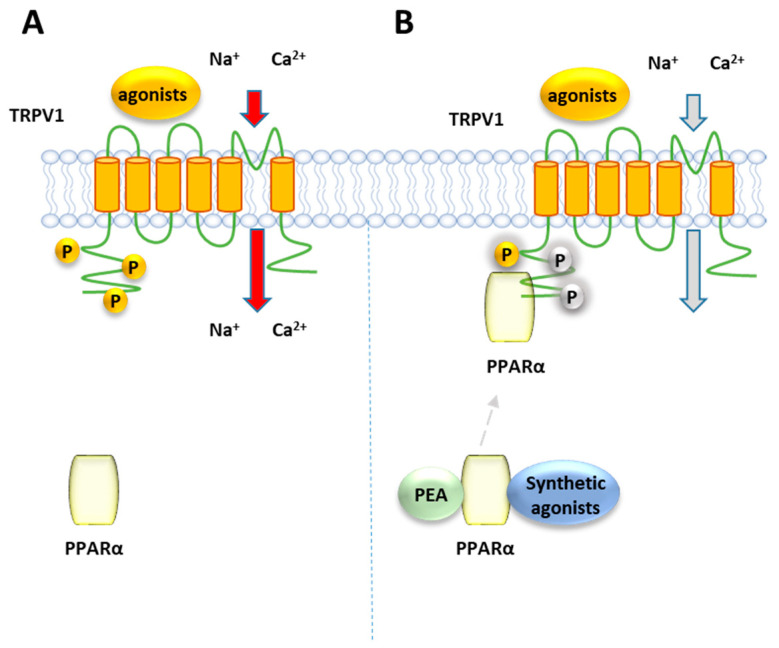
Representative mechanism of TRPV1 receptors regulation by PPARα. (**A**) TRPV1 channels stimulation induced by agonists. Phosphorylation of TRPV1 (indicated as P in yellow circles) is known to be a cellular event promoting the channel activity (**B**) The activation of PPARα by PEA or canonical agonists, suppresses TRPV1 activity, making the channels refractory to stimuli.

Ambrosino et al. [141] have shown that PEA, as well as the canonical PPARα agonists clofibrate (CLO) and GW7647, induce a PPARα-dependent activation and desensitization of TRPV1 currents in TRPV1-transfected CHO cells, where PPARα is endogenously present (Figure 7). While TRPV1 currents induced by capsaicin were unaffected by the PPARα antagonist GW-6471, those evoked by the PPARα canonical agonists and PEA were greatly inhibited. The involvement of the TRPV1 channel in the elevation PPARα-dependent of [Ca^2+^]_i_ was confirmed by the ability of the TRPV1 antagonist capsazepine to counteract the calcium signaling induced by PEA and the other PPARα agonists in sensory neurons. Later, the same authors confirmed this result using CLO, WY14643 or GW7647, differing each other in structural features, potency and selectivity for PPARα over the other two PPAR isoforms γ and β/δ. All these compounds were shown to act as partial TRPV1 agonists in TRPV1-transfected CHO cells, eliciting an efficacy of about half of that of capsaicin [142] and different potency, with CLO and WY14643 resulting about 100-times weaker than GW7647. This rank of potency for TRPV1 activation in vitro is similar to that observed for their analgesic activity in vivo [35]. Moreover, the involvement of PPARα in TRPV1 activation by these compounds was shown by down-regulating or up-regulating the levels PPARα expression: while capsaicin was unaffected by this modulation, a reduction/potentiation of CLO-induced TRPV1 currents was were observed in PPARα-silenced CHO cells and PPARα over-expressing cells, respectively [142]. Moreover, all the tested compounds desensitized TRPV1 to a greater extent than capsaicin. Evidence of a close association between PPARα and TRPV1 arises from co-immunoprecipitation experiments performed on total protein lysates from CHO cells co-expressing TRPV1 channels and enhanced GFP (EGFP)-tagged PPARα receptors, which showed a significant fraction of PPARα in the anti-TRPV1 purified fraction. Since phosphorylation enhances the function of TRPV1 [143], it is possible to speculate that PPARα activates and desensitizes TRPV1 by promoting its phosphorylation, similarly to what occurs with the regulation PPARα-mediated of the nicotine acetylcholine receptors [144].

### 8.2. PPARγ and TRPV1

**Figure 8:**
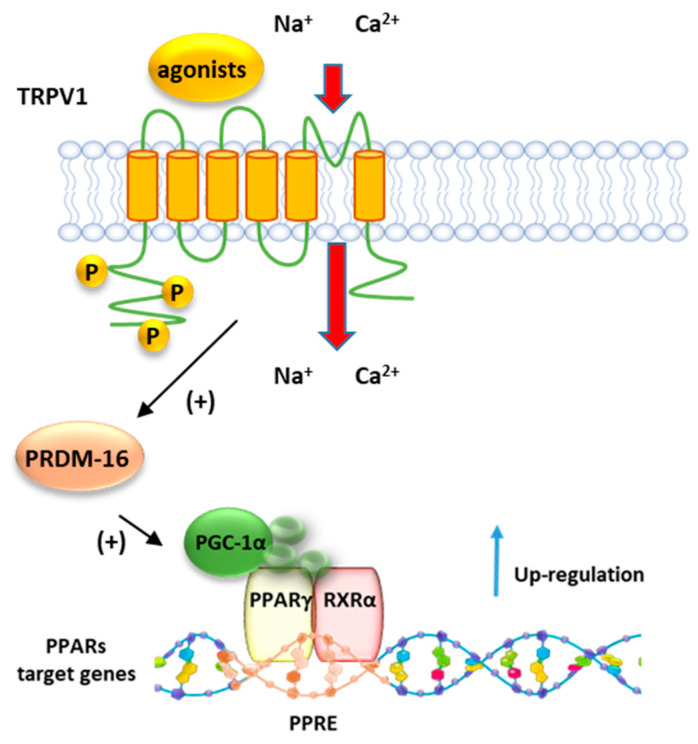
Representative mechanism of regulation PPARγ by TRPV1. Following the activation by agonists, TRPV1 promotes the activity of PRDM16 which in turn induces the activation of PGC1α, a major co-activator of the PPRA/RXR complex.

Baskaran et al. demonstrated that dietary capsaicin enhances the expression levels of PPARγ and PGC-1α (Figure 8) in wt but not in Trpv1 KO mice (Figure 8). Moreover, it stimulates the SIRT-1-dependent deacetylation of PPARγ, which triggers the browning of white adipose tissue (WAT) and prevents the obesity induced by a high-fat diet in mice [145]. The browning of WAT is a mechanism by which energy expenditure is promoted via thermogenesis. Dietary administration of capsaicin increases the transcription factor PRDM-16 and facilitates its interaction with PPARγ (Figure 8), further contributing to inducing browning of WAT.

### 8.3. PPARδ and TRPV1

The study carried out by Li et al. [146] showed that TRPV1 activation reduces lipid accumulation in hepatocytes by stimulating lipolysis without affecting lipogenesis. This effect occurs through the activation of PPARδ since chronic administration of capsaicin promotes the up-regulation of the hepatic PPARδ expression level and the enhancement of PPARδ-mediated hepatocyte autophagy in wt but not in TRPV1 KO mice, thus disclosing the beneficial role of chronic dietary capsaicin in the prevention of hepatic steatosis, inflammatory responses, and body weight gain. The cross-talk between PPARδ and TRPV1 also emerged in a study carried out by Gao et al. [147] where the administration of chronic dietary capsaicin attenuates cardiac hypertrophy and fibrosis associated to with a high-salt diet through the TRPV1-mediated up-regulation of PPARδ expression in cardiomyocytes.

## 9. Conclusions

PPARs represent relevant therapeutic targets for neurodegenerative, metabolic, and inflammation-related disorders. On the other hand, several reports have highlighted the therapeutic potential of (endo)cannabinoid molecules for the same diseases. Thus, the ability of such lipid molecules to either directly activate PPARs or modulate them through the cross-talk between PPARs and cannabinoid receptors opens new avenues for the development of synergistic therapeutic approaches. Additionally, there is fascinating evidence that perturbation of the symbiotic gut -microbial community may have a great impact on the host health either directly or indirectly by regulating PPARs activity through new endogenous, endocannabinoid-like modulators. In this context, it has been recently demonstrated that daily ingestion of *Akkermansia muciniphila*, a mucin-degrading bacterium commonly found in the human gut, reduces the risk of developing diet-induced obesity and concomitantly insulin resistance by increasing the plasma levels of mono-palmitoyl-glycerol, an endocannabinoid lipid acting as PPARα agonist [148]. Conversely, deletion of the gut microbiota in germ-free mice is accompanied by a strong increase in PPARα expression and PPARα NAE ligands in the small intestine [149]. Therefore, the identification of novel modulators and mechanism regulating PPARs activity could pave the way towards future healthcare strategies and therapeutic solutions.
